# LCA and negative emission potential of retrofitted cement plants under oxyfuel conditions at high biogenic fuel shares

**DOI:** 10.1038/s41598-022-13064-w

**Published:** 2022-05-27

**Authors:** Otavio Cavalett, Marcos D. B. Watanabe, Kristina Fleiger, Volker Hoenig, Francesco Cherubini

**Affiliations:** 1 Department of Energy and Process Engineering, Industrial Ecology Programme, Norwegian University of Science and Technology (NTNU), Høgskoleringen 1, 7491 Trondheim, Norway; 2 VDZ gGmbH, Toulouser Allee 71, 40476 Düsseldorf, Germany

**Keywords:** Carbon capture and storage, Renewable energy, Environmental impact, Climate change

## Abstract

The implementation of oxyfuel carbon capture and storage technologies in combination with use of alternative fuels comprising high biogenic shares is promoted as an attractive climate change mitigation option for the cement sector to achieve low or even negative carbon emissions. Here, we perform a prospective life cycle assessment of two state-of-the art cement plants, one in Sweden and one in Germany, under conventional and retrofitted oxyfuel conditions considering alternative fuel mixes with increasing bio-based fractions of forest residues or dedicated bioenergy crops. The analysis also considers effects of the projected changes in the electricity systems up to 2050. Retrofitting the cement plants to oxyfuel reduces climate change impacts between 74 and 91%, while with additional use of biomass as alternative fuel the cement plants reach negative emission between − 24 and − 169 gCO_2eq._ kg_clinker_^−1^, depending on operational condition, location, and biomass type. Additional emission reduction of − 10 (Sweden) and − 128 gCO_2eq._ kg_clinker_^−1^ (Germany) are expected from the decarbonization of the future electricity systems. Retrofitting the cement plants to oxyfuel conditions shows trade-offs with other environmental impacts (e.g., human toxicity, water and energy depletion), which are partially offset with projected changes in electricity systems. Our results illustrate the large climate change mitigation potential in the cement sector that can be achieved by the implementation of oxyfuel carbon capture and storage and biomass use as alternative fuel.

## Introduction

Concrete is the world’s most widely used man-made material. With an estimated yearly consumption close to 30 billion tons, concrete outpaces production of any other material. Even with an expected increase in the use of other building materials (e.g., wood^1^) the global cement demand is projected to continue expanding^2^. The essential ingredient of concrete is cement. Cement production is currently responsible for approximately 6–8% of the global anthropogenic CO_2_ emissions and about 3% of energy use, and projections indicate a 50% increase in annual production volumes by 2050^2,3^. The cement sector faces the challenge of meeting an increasing demand while cutting CO_2_ emissions from its production. According to a recent roadmap form the IEA, in order to be consistent with the Paris Agreements, CO_2_ emissions from cement manufacture need to fall by at least 16% by 2030 and by 24% by 2050, despite the expected increase in demand^4^.

A special characteristic of the cement industry is that about two thirds of the emitted CO_2_ is generated from the chemical reactions involved in converting limestone to calcium oxide, which is a precursor to the formation of clinker phases giving the cement its properties. The remainder one third of the climate impacts are related to emissions from combustion of fuels (usually fossil-based) and other downstream plant operations^4,5^. Therefore, more conventional carbon mitigation measures often proposed for other industrial sectors like energy efficiency improvements^6^, use of alternative fuels^7^ and increasing materials substitution^8^ can only help to decrease the emissions associated with a small share of climate impacts (about one third). For this reason, significant overall emission reductions in line with global climate stabilization targets are likely to be only achieved with the integration of emerging and innovative technologies like carbon capture and storage (CCS)^9–13^. CCS is thus expected to provide the largest cumulative CO_2_ emissions reductions in the cement industry by 2050^4,11^. CCS options for cement sector include amine scrubbing^14^, oxyfuel combustion^15^, calcium looping^16^, and membranes^17^ and their relative advantages and challenges have been broadly discussed in recent years (e.g. refs.^10,12,18^). In this context, CCS using oxyfuel technologies is particularly attractive as the oxyfuel combustion conditions in the kiln with new oxyfuel burner concepts facilitate both the capture of CO_2_ and the use of higher shares (e.g., > 20%) of biomass as alternative fuel. The oxyfuel technology offers the benefit of a variable oxygen use, which can enhance the ignition and fuel burn-out^19,20^. Therefore, the combination of oxyfuel CCS technologies with alternative fuels at high biomass content is an attractive option to achieve large rates of decarbonization in the cement sector.

Climate change is only one of the many sustainability challenges our society is facing. There are several other important environmental issues that are connected with the cement manufacturing process including, for example, contributions to air pollution and consequent human health impacts^3^, depletion of fossil^14^, material^9^, and water resources^21^. Addressing only one of these issues may cause unintended adverse effects in the other environmental areas and lead to suboptimal sustainability strategies. A comprehensive assessment of the various relevant environmental issues is instrumental to unravel potential sustainability trade-offs before large-scale deployment of novel climate mitigation technologies in the cement sector.

The implementation of oxyfuel technologies have been increasingly studied in the recent years, including technical aspects of the oxyfuel burner^15,19,22^, economic viability^23–25^, carbon emissions at stake in comparison with alternatives^5,26,27^, as well as synthesis reports about the potentials, limitations and applicability of this technology in the cement industry^10,12^. In general, these studies assert the relevant climate mitigation potential of oxyfuel CCS technologies in the cement sector, as well as its potential to improve process fuel efficiency and the relatively lower costs in comparison to alternative CCS options. At the same time, they highlight some drawbacks such as the need for additional electricity demand and substantial re-engineering and rebuild of many parts of the cement production process to minimize air ingress and maximize heat recovery^28^. A few studies included other environmental impacts than climate change when evaluating oxyfuel technologies in the cement sector, e.g. ref.^29^, but these are still modeled considering idealized cases. Environmental sustainability analyses of retrofitted oxyfuel plants using real world operation conditions^15,30^, and the integration of oxyfuel technologies with high shares of biomass-based alternative fuels^27^, have not been yet jointly investigated.

In this study, we perform a prospective life cycle assessment of retrofitted cement plants to oxyfuel conditions with CCS based on both real operational and process modelling data for two representative cement plants, one located in Germany and one in Sweden. We analyze the implementation of oxyfuel CCS technology in combination with higher shares of biomass from both dedicated bioenergy crops and forestry residues as alternatives fuel to quantify the potential for achieving negative emission in the cement plants. Their performance is benchmarked against a reference cement plant with typical European data. Our analysis applied updated life cycle assessment methods to address synergies and trade-offs between climate change effects (also considering different metrics and time horizons with and without the inclusion of near-term climate forcers) and other key environmental impacts categories, namely human health, energy and water depletion. Projections for future background electricity supply systems are explicitly embedded in our analysis to address the influence of these changes on the environmental performance of the oxyfuel CCS cement plants.

## Methods

### Life cycle assessment

Life cycle assessment (LCA) aims to quantify potential environmental impacts throughout a life-cycle of a product, process or service, including direct and indirect emissions and use of resources from raw material acquisition through production, use, end-of-life treatment, recycling and final disposal^31^. The method has evolved over the last three decades to become a central tool for environmental management and decision support, in particular serving as scientific basis for policies and plans, consumer information and public procurement^32,33^.

**Figure 1:**
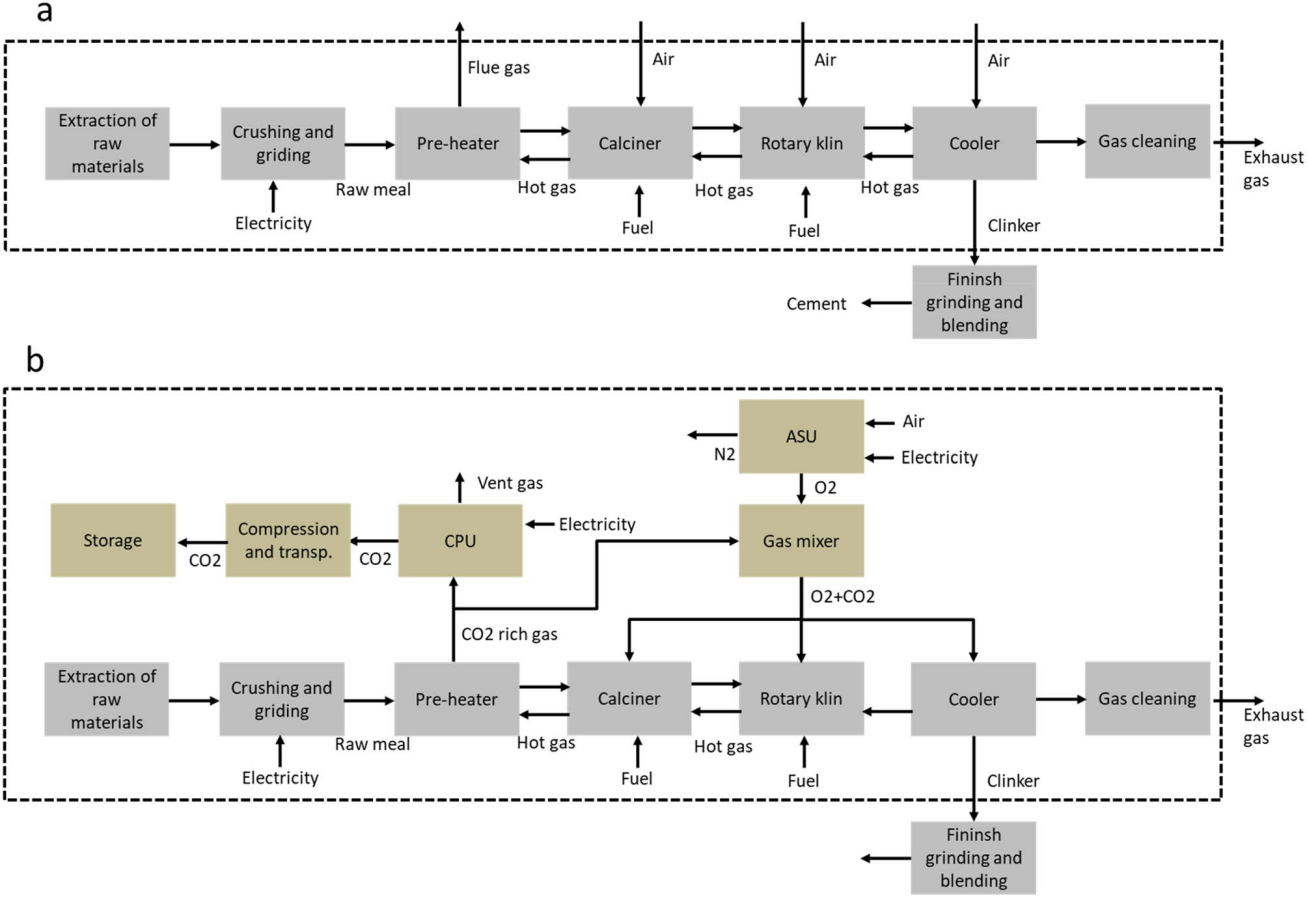
Main stages of the conventional cement production process (**a**) and retrofitted cement plants for oxyfuel combustion and CO_2_ capture (**b**). Product systems are indicated inside the dashed boxes. ASU: air separation unit; CPU: CO_2_ purification unit. Adapted from^4,19^.

The objective of our study is to assess two real-world cement plants retrofitted to oxyfuel CCS conditions in terms of selected environmental impacts. The analysis includes the effects of increasing use of biomass as alternative fuels and prospective future energy systems in the environmental impacts. The scope considers a cradle-to-gate analysis and includes the raw material acquisition, material and fuel transportation stages and the clinker production including energy and material inputs and emissions to the air from the cement plant (Fig. 1). The life-cycle stages related to clinker finishing, grinding and cement formulation are not included as they are expected to be the same for all the cases. The functional unit is 1 kg of clinker, the key ingredient of cement products. Clinker content in cement products corresponds to more than 95% (in mass) in Portland cement and varies depending on the intended product application. Foreground data are based on plant operational data and process simulation results. Background life cycle inventory systems are retrieved from ecoinvent 3.6 database^34^. Projections for future electricity systems are incorporated into a forward-looking background database and are based on outputs from Integrated Assessment Models (IAMs) (see specific section about this topic in the Methods). The analysis focuses on four selected environmental impact categories, namely climate change, human toxicity (HT), fossil depletion potential (FDP) and water depletion potential (WDP).

Most of climate change studies using LCA widely rely on the 100-year global warming potential (GWP100) as the default emission metric for addressing climate change impacts. The shortcomings of this approach have been discussed in the recent years^35–38^. To overcome them, a multimeric approach has been frequently recommended^37^. This approach is based on the complementary use of Global Warming Potential (GWP) and Global Temperature change Potential (GTP) for different time horizons for addressing the distinct implications of emissions in the climate system. Climate metrics are usually sensitive to the time scale of climate forcers, especially for those species with atmospheric lifetimes substantially shorter than that of CO_2_. For example, while most of the CO_2_ emissions stay in the atmosphere on millennial time scales^39^, many near term climate forcers (NTCF), such as CO, NOx, SOx, volatile organic compounds (VOC), black carbon (BC) and organic carbon (OC), leave the atmosphere in a few days or months after emissions. This means that NTFCs are not well mixed in the atmosphere and can result in regional impacts that differ from the global average, depending on regions where they are emitted^40^. GWP is used with two time-horizons to capture short-term (GWP20) and medium-term dimensions (GWP100) of the climate system response and GTP100 is used as a proxy for long-term impacts. In the analysis with GWP20, NTCFs are assessed with both globally averaged metrics and also metrics specifically developed for Europe to better represent their regional effects. GWP100 is considered a proxy for mid-term impacts because of the numerical similarity between GWP100 and GTP40 (i.e., GWP100 can basically be interpreted as a metric indicating temperature impacts at about four decades after an emission)^41^ On the other hand, GTP100 specifically quantifies impacts 100 years after an emission and it is a suitable metrics to assess contributions to long-term temperature stabilization objectives^38^. Characterization factors are taken from IPCC Fifth Assessment Report^42^ and are shown in the Supplementary Table 1.

HT considers the potential impacts from the emission of air pollutants (e.g., NOx, SOx and particulates) and heavy metals to the air, soil and water in people’s cancer and non-cancer incidence rates via fate, exposure and damage factors. FDP and WDP account for the use of energy resources and water, respectively, in all the stages of the production life cycle. Characterization factors for these three environmental impact categories are from the ReCiPe life cycle assessment method^43^, one of the most used and updated methods in LCA.

### Cement plant process data

Data for the two selected cement plants are modeled using a comprehensive process engineering model built by VDZ^44–49^, as part of the AC^2^OCem Project results^50^. The model is capable to realistically represent the clinker burning process. At its core, it describes the process from the kiln meal feed to the outlet of the clinker from the cooler and it is made up of individual models for the plant components preheater, calciner, bypass, rotary kiln and grate cooler. All the individual model sections can be linked mathematically with one another, which makes it possible to determine a steady-state condition for the entire clinker production process. The calculations themselves cover the energy and material balances. The combustion calculations for the fuels and the heat transfer, the relevant chemical and mineralogical solid-state reactions, the gas phase reactions, and the gas-solids reactions are taken into account. The model has been further developed to also represent the oxyfuel technology with flue gas recirculation^49^ and has been refined by results from prototype tests within the CEMCAP project^51^. Further burner pilot tests in AC2OCem project provide additional data on the combustion characteristics when applying alternative fuels, which are included in the refined oxyfuel model. Life cycle inventories are then produced using both real-world operational data and the modelled mass and energy balances. The life cycle inventories for the two cement plants operating under conventional and oxyfuel condition with different biomass use as alternative fuels are summarized in Supplementary Tables 3 and 4.

### Cement plants selected for retrofitting with CCS oxyfuel technology

Oxyfuel technology for cement plants has been studied theoretically for many years under idealized situations^14,27–29,52^. However, each cement plant has its specific conditions, which require tailored investigations for applicable and enhanced oxyfuel retrofit design. Therefore, the implementation of oxyfuel technology in existing cement plants demands establishment of specific retrofit requirements to be performed considering boundary conditions of the plant, including, for instance, energy efficiency aspects, fuel characteristics, raw meal quality, specific emissions, plant capacity, capacity of key aggregates and on-site space for additional equipment.

In this study, two existing European cement plants were selected for quantifying the environmental implications of retrofitting them with oxyfuel technology. These two plants (plant A in Germany, plant B in Sweden) are selected as they show different site-specific boundary conditions (e.g. raw material moisture, electricity consumption), which influences the process technology and the layout of the oxyfuel retrofit. Both of them already use a high share of alternative fuels, e.g., > 75%, mostly refused derived fuels (RDF) mixed with fossil fuels (e.g. coal and lignite). Information regarding the fossil and alternative fuels used is the cement production cases are presented Supplementary Tables 2 and 6. The production capacity of plant A is 4440 tons of clinker per day and plant B produces 5765 tons of clinker per day. A complete summary of the key inputs and outputs of the two cement plants is provided in the life cycle inventories (LCI) in Supplementary Tables 3 and 4. The information about processing efficiencies, use of inputs and emissions of pollutants are based on real world operational data obtained from the plant operational data reports and also validated using computer process simulation.

### Reference cement plant

To benchmark the results of the two cement plants, a reference case (REF) was selected considering a modern cement plant with an average European cement plant technology proposed by the Cement sustainability Initiative (CSI) and European Cement Research Academy (ECRA)^53^. In general terms, this plant has a processing capacity of 3000 tons of clinker per day and considers a dry kiln process, consisting of a five-stage cyclone preheater, calciner with tertiary duct, rotary kiln, and clinker cooler. Emissions of air pollutants included both combustion of fuel in the calciner and the rotary kiln, as well as from the calcination of the raw material itself. A selective non-catalytic reduction (SNCR) system for NOx removal is included in the process configuration^14,23^. The thermal and the electric power consumption of the plant is 3380 kJ kg_clinker_^−1^, and 0.132 kWh kg_clinker_^−1^, respectively. The thermal energy is supplied using hard coal as fuel and electricity input considers the average European mix. Complete LCI data is presented in the Supplementary Table 5. This reference case and additional benchmark case operating with oxyfuel combustion CO_2_ capture is described in other studies^14,23,53^.

### Oxyfuel combustion and CO_2_ capture, transport and storage

The oxyfuel CO_2_ capture technology is based on combustion of the fuel in an atmosphere of oxygen and recirculated flue gas (mainly CO_2_) instead of air. With the use of oxyfuel combustion, the cement kiln process itself must be modified. The gas atmosphere in the clinker cooler, the rotary kiln, the calciner and the preheater is changed. The flue gases are then ideally composed of water vapour and CO_2_, which are easily separated by condensation, as compared to a conventional combustion with a post-treatment capture scheme, where CO_2_ requires energetically intensive chemical separation^15^. In oxyfuel combustion, flue gas recirculation is essential to control the temperature in the kiln and to provide suitable gas velocities to the cement process^19^. Even if oxyfuel technology does not necessarily incur additional fuel consumption, the process requires re-engineering of the plant to optimize the heat recovery system and minimize air ingress. To integrate the oxyfuel technology into the clinker burning process, additional power is considered for the oxygen supply facility (ASU) (i.e. 0.2 kWh kgO_2_^–1^) and a CO_2_ purification unit (CPU) (i.e. 0.154 kWh kgCO_2_^–1^) to enrich the CO_2_ stream and allow its transport and storage^29^. Both these additional plant components significantly influence the electricity consumption. The CPU is designed as a single flash, self-refrigerated unit which delivers compressed CO_2_ at 110 bar. The CPU includes compression with intercooling in four stages and drying of the CO_2_ stream with molecular sieves prior to cooling and liquefaction of CO_2_ in a multi-stream heat exchanger^14,25^.

The transportation and storage locations and energy demands for the captured CO_2_ are modelled according to ref.^54^. The CO_2_ is considered to be stored in the Johansen formation (northern part of the North Sea), in line with the NorthernLights project^55^. This avoids the regulatory limitations for on-shore CO_2_ storage in some countries, including Germany. Half of CO_2_ is considered to be transported by ships (100 kWh/ton of CO_2_) and half by pipeline (84.8 kWh/ton of CO_2_). In our analysis, a carbon capture efficiency of 90% is assumed, in line with recent studies of oxyfuel technology^14,23^ This takes into account the intrinsic capture process technical limitations and CO_2_ leakages from transport and storage media.

### Use of alternative fuel with high biogenic shares

For several years the cement industry has been using waste-based fuels containing biomass. The substitution rate is constantly increasing and was approximately 31% in Europe in 2019^56^. Biomass (such as wood chips and pellets, sewage sludge, animal meal, etc.) and other alternative fuels as refused derived fuels (RDF) from industrial and public wastes with high share of biogenic materials are particularly interesting alternative fuels for the cement industry, as they can largely contribute to reduce the fossil carbon emissions from the clinker burning process and, in combination with carbon capture and storage technologies (BECCS), may allow very low or even negative emissions in the cement manufacturing in comparison to the conventional process^57^. It happens because the biogenic carbon is removed from the atmosphere during the plant growing and it is stored in biomass. Therefore, when biomass is combusted and these carbon emissions are captured and stored, it is equivalent to a removal of carbon from the atmosphere (or a negative emission). This is different when CCS is applied to carbon emission for fossil fuels, which are instead avoiding that the carbon in fossil resources is released into the atmosphere.

In principle, cement kilns can utilize up to 100% of alternative fuels. However, the alternative fuel share in conventional clinker burning process may be capped to amounts that provide high enough temperature in the rotary kiln in addition to some other technical limitations. An important feature of the CCS using oxyfuel technology is the fact that the oxyfuel combustion conditions may allow the use of higher shares of biomass as alternative fuel, as the variable use of oxygen enhances the fuel burn-out and ignition of the fuels. In the present study the conventional clinker burning process is modelled using higher shares of biomass in the fuel mix by proportionally increasing the biomass shares until the lower heating value (LHV) of the average fuel mix in the calciner is at least 15 MJ kg^−1^ (and 22 MJ kg^−1^ in the main firing of the cement kiln), as indicated by ref.^53^. The resulting fuel shares and LHV of the fuel mix in the different process alternatives are indicated in Supplementary Table 6. While increasing the biomass shares, all the other fuels used in the clinker burning process are proportionally reduced. An additional theoretical case was considered where the remainder fraction of fossil fuel (coal and lignite) used in the two cement plants is also replaced by biomass, therefore reaching 100% use of alternative fuels under oxyfuel conditions. While mass and energy balances indicate that it is possible to achieve the required temperature in the kiln process, these are extreme cases used for illustrative purposes, still without experimental support for its implementation.

The biomass options considered as alternative fuel for the cement clinker production are miscanthus and forest residues. In Europe, miscanthus generally shows good yields and the ability to adapt to a wide variety of climate and soil conditions and can easily be incorporated into existing farming systems as conventional agricultural machinery can be used. We consider an LCI that includes all the agricultural operation under rain feed conditions and inputs from crop establishment, fertilization, harvesting and transport to the cement plant^58^. Biomass from forest residues are also included to represent the ambitions to prioritize the available residual biomass resources as a strategy to stimulate a circular economy perspective, prevent additional pressure on terrestrial ecosystems, and provide an enhanced use of the available residual streams. The modelling of biomass potential from forest residues is based on previous studies on potentially sustainable removal rates^59–61^. The complete biomass value chain is modelled to account for inputs and emissions related to silviculture, harvesting, transport, chipping, processing, and transport of forest residues to be used in the cement sector. Sector-specific data for forestry operations and logistics are used, which are detailed elsewhere^61^. A list of the considered cement production cases is presented in Table 1 and a summary of the key characteristics of these cases is presented in the Supplementary Table 6.

**Table 1:** Cement production cases considered in this study.

Cement production case	Description
REF	Cement production considering modern process technology based on the average cement sector in Europe
REF Oxy	Same as REF but considering the plant retrofitted to oxyfuel operational conditions
Plant A	Plant A operating in Germany under current (real world) operational conditions
Plant A Oxy	Plant A retrofitted to oxyfuel operational conditions
Plant A B(M)	Plant A operating under conventional (air) conditions and a higher share of biomass from miscanthus as alternative fuels
Plant A OxyB(M)	Plant A retrofitted to oxyfuel operational conditions using a higher share of biomass from miscanthus as alternative fuels
Plant A OxyB(M) +	Plant A retrofitted to oxyfuel operational conditions using 100% biomass from miscanthus as alternative fuel
Plant A B(FR)	Plant A operating under conventional (air) conditions and a higher share of biomass from forest residues as alternative fuels
Plant A OxyB(FR)	Plant A retrofitted to oxyfuel operational conditions using a higher share of biomass from forest residues as alternative fuels
Plant A OxyB(FR) +	Plant A retrofitted to oxyfuel operational conditions using 100% biomass from forest residues as alternative fuel
Plant B	Plant B operating in Sweden under current (real world) operational conditions
Plant B Oxy	Plant B retrofitted to oxyfuel operational conditions
Plant B B(M)	Plant B operating under conventional (air) conditions and a higher share of biomass from miscanthus as alternative fuels
Plant B OxyB(M)	Plant B retrofitted to oxyfuel operational conditions using a higher share of biomass from miscanthus as alternative fuels
Plant B OxyB(M) +	Plant B retrofitted to oxyfuel operational conditions using 100% biomass from miscanthus as alternative fuel
Plant B B(FR)	Plant B operating under conventional (air) conditions and a higher share of biomass from forest residues as alternative fuels
Plant B OxyB(FR)	Plant B retrofitted to oxyfuel operational conditions using a higher share of biomass from forest residues as alternative fuels
Plant B OxyB(FR) +	Plant B retrofitted to oxyfuel operational conditions using 100% biomass from forest residues as alternative fuel

### Future background electricity systems

One major shortcoming of many LCA addressing novel technologies is the reduced capacity to embed the evolution of background life-cycle inventories for the key inputs that are representative of different projections in terms of sectoral transformations, such as future electricity systems, despite the ongoing substantial decarbonization trends with increased shares of renewable energy sources. These changes are expected to be relevant for the cement plants assessed in this study, which should become operational within a few years when the background electricity mixes can be different than today. Recent efforts attempt to cover this gap have been proposed by the so-called prospective LCA, where projections from Integrated Assessment Models (IAMs) are integrated within an LCA framework^62–64^. A proper representation of these changes in the energy system is therefore crucial in LCA studies of developing cement clinker production alternatives considering the implementation of forward-looking technologies. As oxy-fuels conditions typically increase electricity consumption, the electricity systems may be an important factor for the environmental impacts of retrofitted cement plants.

To account for the influence of technological changes that are projected to occur in the future energy systems, the *premise* Python codes (version 0.4.2)^65^ is used to generate new background life cycle inventories databases (ecoinvent 3.6) for the electricity production technologies considering the output results of REMIND Integrated Assessment Model^66^. These new life cycle inventories adjust the representation of technological conditions of the electricity supply options under future policy scenarios by transforming the electricity production mixes and power plants efficiencies considering the next three decades: 2030, 2040, and 2050 under a specific shared-socio-economic pathway (SSP) known as ‘Middle of the Road’. In SSP2 narrative, the world follows intermediate challenges for mitigation and adaptation, with moderate population growth, energy use decline, but slow progress in achieving sustainable development goals^67^. We also selected the climate policy scenario considering the conditions of emission reductions and other mitigation commitments of the Nationally Determined Contributions under Paris Agreement are implemented^65,66^. This scenario is predicted to keep global warming levels below 2 °C by 2100. The new background life cycle inventories databases representing the projected electricity technology options are combined with updated country-specific projections of the electricity sector considering projections aiming at the long-term gradual implementation of increased shares of renewable energy. Data for Germany^68,69^ and Sweden^70^ (see Supplementary Tables 7 and 8) are used as these are the countries where the two cement plants are located. A full life cycle perspective is considered in our analysis, therefore ensuring that all use of resources and emissions of the different electricity production technologies are also included as background system for cement plant inputs.

## Results and discussion

### Environmental impacts of retrofitted cement plants under oxyfuel conditions

**Figure 2:**
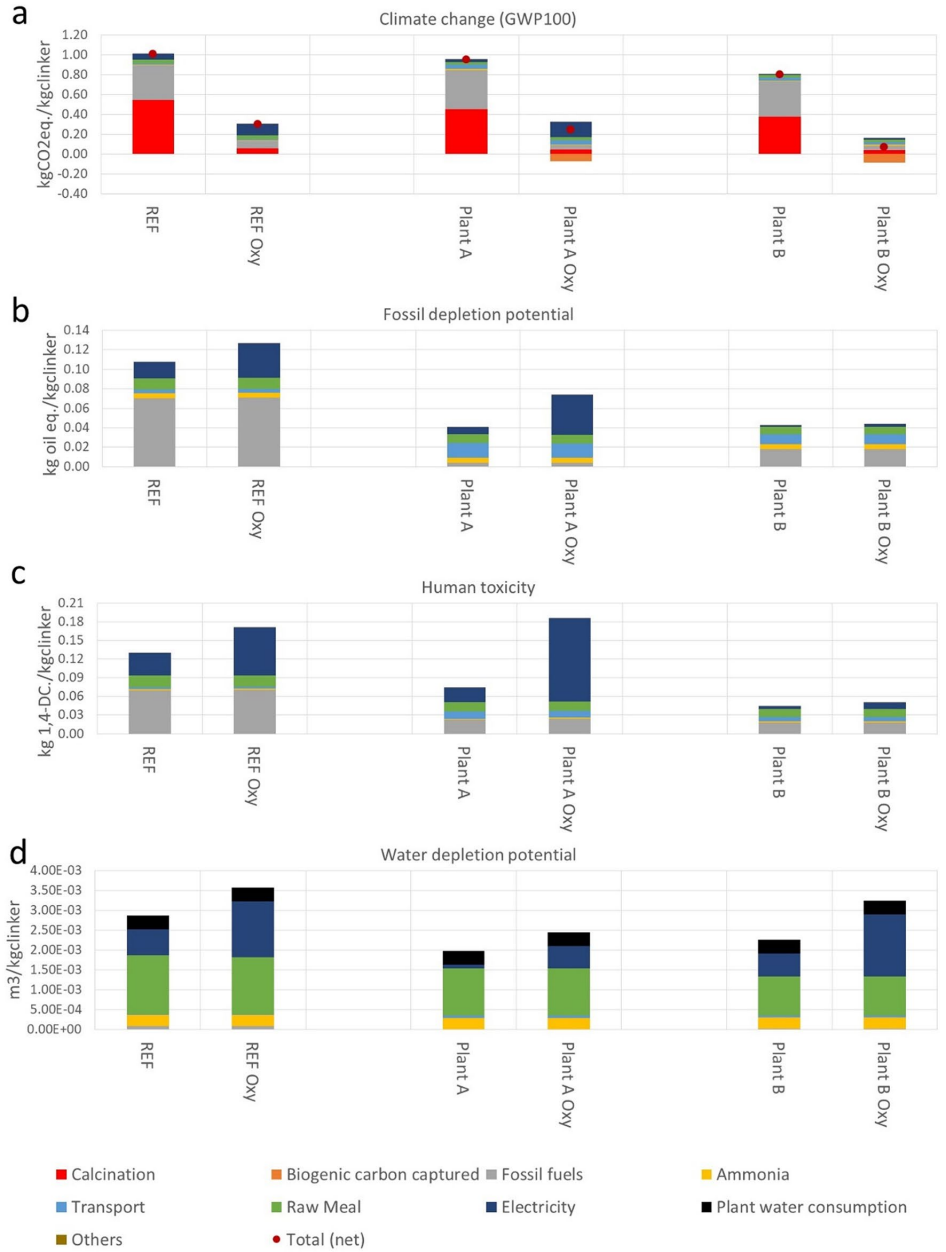
Selected life cycle environmental impact categories for the cement clinker production cases under conventional and oxyfuel CCS (Oxy). Impacts on climate change (GWP100) (**a**), fossil depletion potential (FDP) (**b**), human toxicity (HT) (**c**), and water depletion potential (WDP) (**d**). Plant A is located in Germany plant B in Sweden REF represent a reference plant based on average European cement technology.

The breakdown of environmental impacts of the different cement clinker production cases operating under conventional and oxyfuel conditions are presented in Fig. 2. Retrofitting the cement plants to oxyfuel conditions reduces the climate change impacts (GWP100) by 74% for plant A (located in Germany) and 91% for plant B (located in Sweden), while this reduction is 70% for REF case (Fig. 2a). Under oxyfuel conditions, there is an increase in the contribution from electricity for the ASU and CPU units. When retrofitted to oxyfuel conditions, the relative contribution from electricity use in the climate impacts increases from 5 to 38% in the REF plant, from 3 to 63% in the plant A, and from 1 to 19% in the plant B. Plant A and B present a sizeable biogenic carbon captured by CCS due to the biogenic shares in the current use of alternative fuels. The biogenic carbon captured corresponds to 24% and 56% of the total other process emission in plant A and plant B, respectively (see Supplementary Tables 3 and 4 showing the fuel use in each cement production case). As the REF case only uses coal as fuel, there is no biogenic carbon captured and all the sequestered carbon by CCS is from fossil sources. The relative climate impacts form calcination is responsible for 47–54% of the net total climate change impacts in the three cement plants operating under conventional conditions. The net carbon emissions of the plants operating under oxyfuel condition is 244 gCO_2eq._ kg_clinker_^−1^, 71 gCO_2eq._ kg_clinker_^−1^ and 304 gCO_2eq._ kg_clinker_^−1^ for plant A, plant B and REF cases, respectively. Plant B benefits form the remarkably low carbon intensity of the electricity mix in Sweden in comparison with Germany (Plant A) and average Europe (REF), where a considerable share of the electricity mix still relies on non-renewable energy options.

The climate change impacts of the cement clinker production cases vary depending on the climate metric and time horizon considered for the analysis, as shown in the Supplementary Fig. 1. However, the relative climate impact among the different cement clinker production cases remains largely unchanged. This is because these impacts are highly driven by CO_2_ emission, with relatively little contributions of non-CO_2_ greenhouse gases (GHG) and near-term climate forcers, as shown in the Supplementary Table 9. For example, the relative contribution of CO_2_ in the total climate impact varies form 99% in plant A using GTP 100 to 44% in plant B using GWP20.

While climate impacts are largely reduced in the retrofitted cement plants to operating with oxyfuel conditions, the higher use of electricity causes an increase in other impact categories. Impacts on fossil depletion potential (FDP) are 83% and 4% higher for plant A and plant B, respectively (Fig. 2b). In human toxicity (HT) (Fig. 2c), impacts are 153 and 13% higher under oxyfuel conditions and 24% and 43% higher for water depletion potential (WDP) (Fig. 2d) for plant A and plant B, respectively. These differences are mostly associated to the electricity production mixes in the two different countries, as the German electricity mix presents substantial shares of hard coal and lignite, while the Swedish mix is largely based on hydropower and nuclear. For example, the relative contribution form electricity use in the HT impacts (Fig. 2c) increases from 28 to 45% in the REF plant, from 31 to 72% in the plant A, and from 8 to 19% in the plant B when retrofitted to oxyfuel conditions. Increase in WDP impacts (Fig. 2d) due to electricity use in the oxyfuel cases in plant B are mostly due to the considerable share of hydropower in the Swedish electricity mix. Given this remarkable importance of the electricity mixes for the environmental profile of the retrofitted cement plants, the impacts of future energy system changes are explored in a following section.

### Increased use of biomass as alternative fuel

**Figure 3:**
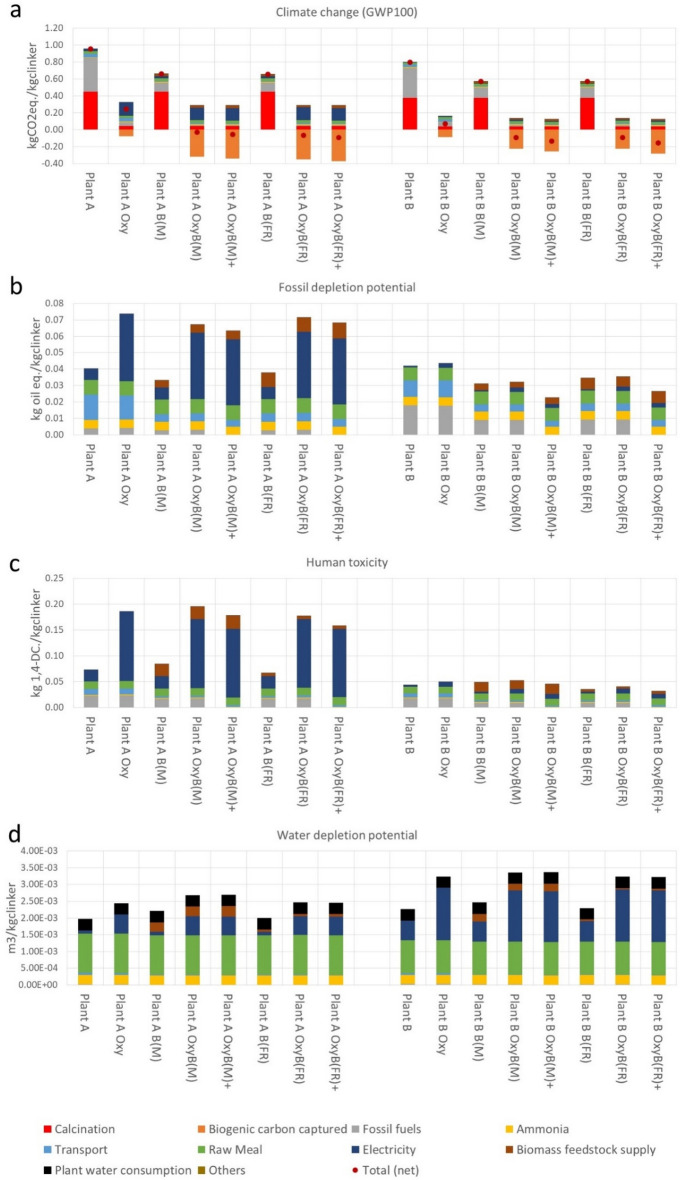
Selected life cycle environmental impact categories for the cement clinker production cases under conventional and oxyfuel CCS (Oxy) with increased use of biomass fuels. Cases indicated with (M) consider biomass supply from miscanthus and (FR) from forest residues. The symbol “ + ” indicates the cases where additional biomass is used to reach 100% of alternative fuels. Impacts on climate change (GWP100) (**a**), fossil depletion potential (FDP) (**b**), human toxicity (HT) (**c**), and water depletion potential (WDP) (**d**). Plant A is located in Germany plant B in Sweden REF represent a reference plant based on average European cement technology.

The use of increased biomass shares in the alternative fuel mix promotes crucial reductions in the climate change impacts of the cement plants (Fig. 3a). Both retrofitted cement plants operating under oxyfuel conditions reach negative climate change impacts with increased use of biomass from dedicated bioenergy crops, meaning that the capture and long-term storage of biogenic carbon is higher than the impacts of GHG emissions from all the other life-cycle stages. There is a net climate change mitigation of − 24 gCO_2eq._ kg_clinker_^−1^ for plant A and − 87 gCO_2eq._ kg_clinker_^−1^ for plant B. Important reductions in the climate impacts, between 29 and 31%, are also obtained for the two plants operating under conventional conditions with increased use of biomass as alternative fuels (i.e., 74% of fuels are supplied with biomass in plant A and 66% in plant B, Supplementary Table 6). In the cases with 100% use of alternative fuels, this mitigation increases to − 57 gCO_2eq._ kg_clinker_^−1^ for plant A and − 135 gCO_2eq._ kg_clinker _^−1^ for plant B. For the REF case, reduction in climate change impacts of 20% for the conventional case and 94% for the oxyfuel case are obtained with increased use of biomass as alternative fuel.

Additional cases are explored with use of biomass from forestry residues instead of dedicated bioenergy crops. In general, climate change mitigation is slightly higher with the use of forest residues instead of miscanthus. For example, results in Fig. 3a show that the climate mitigation of using forest residues with 100% use of alternative fuels increases to − 92 gCO_2eq._ kg_clinker_^−1^ for plant A and − 159 gCO_2eq._ kg_clinker_^-1^ for plant B, making the negative emissions 62% higher for the plant A and 18% higher for plant B. In general, the value chain related to procurement and transport of forest residues present climate change impacts roughly 10% lower than miscanthus biomass.

The major restrictions to the use of biomass in cement manufacturing are normally linked to economic factors, need of pretreatment stages, local availability of the resources and the transport costs, which are in fact more restrictive than technical limitations^71^. A sustainable supply of biomass resources from the international markets might be key to secure this biomass availability and cost competitiveness. If sustainable biomass supply is not available at the scale needed, the results showing that cement production can cause negative CO_2_ emissions cannot be realized. To give a perspective of scale of biomass needed, if we consider that all the annual clinker volumes in Germany and Sweden with the oxyfuel technology described here will use 100% residual biomass as alternative fuels (cases OxyB(FR) +), this biomass demand is equivalent to 21% and 5% of the total potential sustainable available agricultural and forest residues^72,73^ in Germany and Sweden, respectively.

Regarding other environmental impacts categories, the increased use of biomass promotes an increase in impacts from biomass production value chain, which are mostly compensated by the reduction in the contribution from fossil fuels. There is also a noticeable reduction in the contribution from transport of the fuels, mostly in FDP (Fig. 3b) and HT (Fig. 3c), as biomass resources are sourced locally. In general, the value chain related to procurement and transport of forest residues in comparison to miscanthus present about twice more impacts in FDP, but roughly 75% lower impacts on HT and WDP. Regarding FDP (Fig. 3b), the shift from miscanthus to forest residues in the cases with 100% use of alternative fuels increases the participation of biomass in the total impacts from 8 to 14% in plant A and from 16 to 26% in plant B. The biomass value chain contribution to the total impacts in HT of cement clinker production process in plant A varies from 15 to 27% with miscanthus and only 4% to 8% from forest residues, while in plant B the contribution varies from 31–42% for miscanthus and 9% -14% for forest residues, depending on the case (Fig. 3c). For WDP (Fig. 3d), the shift from miscanthus to forest residues in the cases with 100% use of alternative fuels decreases the participation of biomass in the total impacts from 12 to 3% in plant A and from 7 to 2% in plant B.

### Influence of future changes in background energy systems

**Figure 4:**
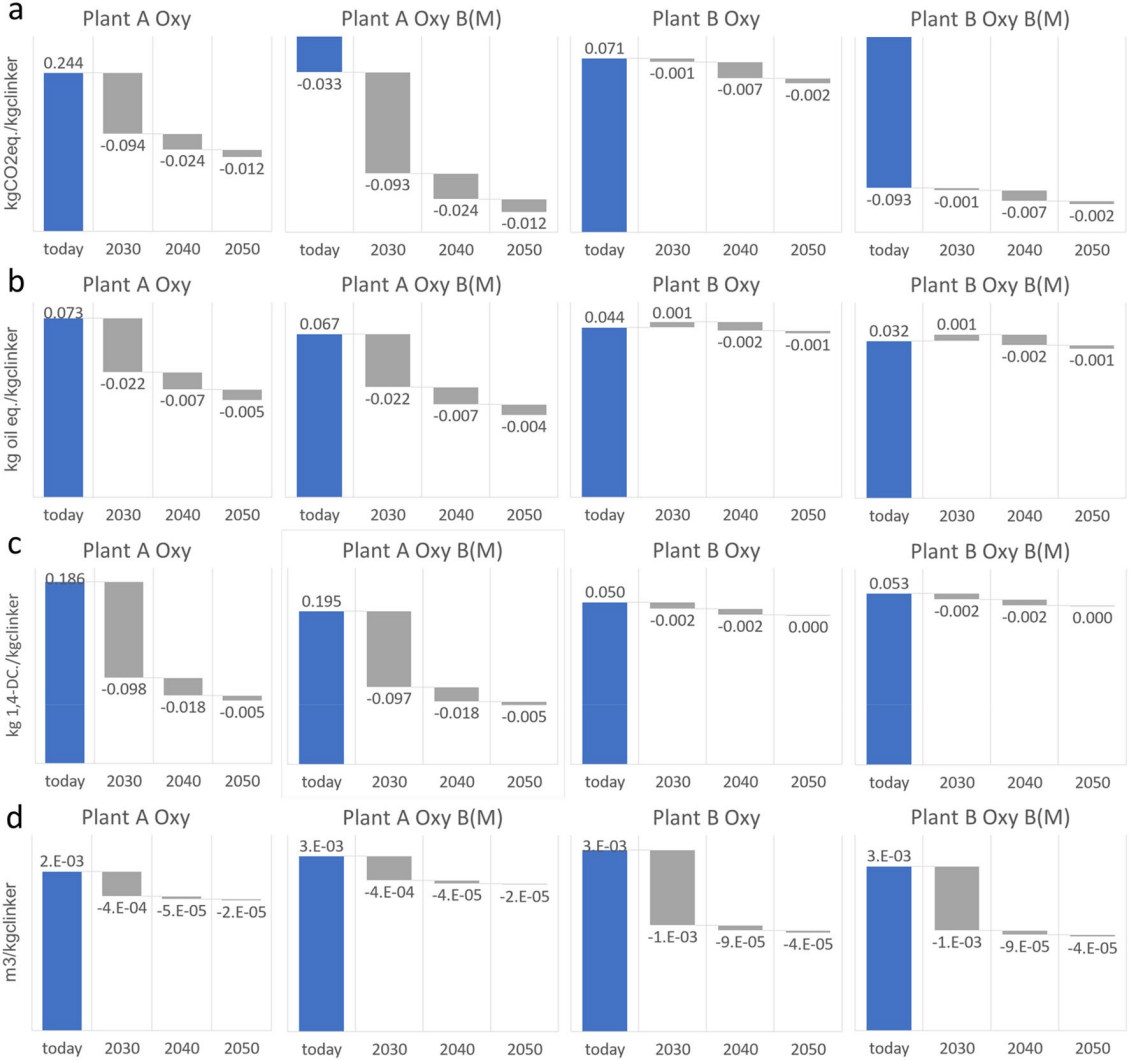
Changes in the environmental impacts of selected oxyfuel CCS cement clinker production with projected future electricity systems. Cases indicates with B(M) consider increased use (66–74% of fuels) of miscanthus biomass as alternative fuel. Impacts on climate change (GWP100) (**a**), fossil depletion potential (FDP) (**b**), human toxicity (HT) (**c**), and water depletion potential (WDP) (**d**). Note the variation of the y axis scales in the different panels.

Results in Fig. 4 show the impacts of projected changes in electricity production technologies according to REMIND Integrated Assessment Model, SSP2-NDC scenario and country specific electricity mix projections in 2030, 2040, and 2050. The cement production options considered are those under oxyfuel conditions without and with increased use of biomass as alternative fuel. These cases are selected because are future-oriented and have higher electricity demand with oxyfuel capture technology implementation (and hence are particularly sensitive to the impacts of the electricity supply mix). In general, all environmental impacts are reduced with the projected future electricity mixes that are consistent with the implementation of Nationally Determined Contributions (NDC) under Paris Agreement. Relatively larger reductions in climate change (Fig. 4a), FDP (Fig. 4b) and Human toxicity (Fig. 4c) impacts are observed for the cement plant in Germany up to 2030. This happens because the cement industry in Germany is expected to benefit from substantial projected substitution of carbon intensive electricity production options like coal and natural gas with renewables like wind and photovoltaic (see Supplementary Table 7). For water depletion potential (Fig. 4d), larger reductions are observed for the cement industry in Sweden up to 2030 due to the projected decrease of hydropower and nuclear (and increase in wind power). There are small trade-offs from the projected future changes in the electricity systems in the FDP (Fig. 4b) up to 2030 in Sweden, which are again affected by the increasing share of wind power in the electricity mix. However, after 2030 this increase is compensated by projected changes in the mix of technologies employed in the electricity system (Supplementary Tables 7 and 8).

In general, the magnitude of impact largely varies across time and country, depending on the current and future electricity systems mix for the different locations. For example, climate impacts are projected to be reduced by 53% for the plant A (in Germany) and only 15% and for plant B (in Sweden) in 2050 relative to today. For the cement clinker production cases with increased use of miscanthus biomass, the negative climate impacts are further increased, providing additional mitigation in 2050 of − 128 gCO_2eq_ kg_clinker_^−1^ for plant A and − 10 gCO_2eq_. kg_clinker_^−1^ for plant B, totaling − 162 gCO_2eq._ kg_clinker_^−1^ for plant A and − 103 gCO_2eq._ kg_clinker_^−1^ for plant B.

## Limitations

It is important to highlight some of the limitations of our study. For example, mass and energy balances indicate that it is possible to use 100% of biomass used as fuels in the cement kiln process. However, there are many potential negative effects of high shares of biomass use as alternative fuel on the cement plant operation such as changes in energy efficiency, thermal stability of mineral impurities, increased incrustation formation and impact on clinker quality that are not taken in consideration in our present environmental analysis. Previous research and development projects have shown that oxyfuel conditions are feasible and can be practically implementable in cement kiln burning processes when using coal as fuel^25^. At the same time, oxygen enrichment to enhance the combustion of biomass containing fuels have been adapted to kiln plants in the past. Although the burner technology for oxyfuel combustion has been the matter of several research and development projects from lab-scale up to large-scale pilot facilities in the power sector^12^ and the physics of combustion in a high CO_2_ atmosphere are mainly understood^15^, the application of this technology in the clinker burning process carries new uncertainties. In particular, those regarding the use of high shares of alternative fuels, including biomass and industry residues with high shares of biogenic carbon. For example, the use of non-wooden biomass such as miscanthus may cause unwanted issues in the cement kiln high-temperature combustion process like ash deformation and sintering. In higher shares, it also might affect the clinker properties by introducing different elements from the ash. Another aspect is that when combusting high shares of non-wooden biomass, a flue gas cleaning system using selective catalytic reduction might be required for keeping the NO_x_ emission under acceptable levels for CO_2_ compression. It might also increase ammonia content in the flue gas, but the expected impacts on overall environmental impact results is small. These aspects could refrain the applicability of some biomass options as fuel in the cement sector and these aspects have not been considered in our analysis. In addition, emissions of N_2_O, a powerful climate forcer, may also increase with oxyfuel combustion of biomass and the use NO_x_ reduction systems. These emissions may leave the process as vent gas and are difficult to be predicted or modeled in plants operating under oxyfuel conditions. Therefore, these factors are also not included in the present analysis but could have a contribution to climate change impacts. Some of these aspects are currently under investigation at research and development stage and prototype burner tests have been successfully conducted at pilot scale considering oxyfuel combustion with 100% alternative fuels with high shares of biogenic content, e.g., ref.^20^. The successful combination of oxyfuel technology with high shares of biomass use as alternative fuel will lead to an enhanced CCS set-up for the cement industry that may achieve very low or even negative emissions in the cement production process.

The use of available biomass residues can be seen as a strategy to stimulate a circular economy perspective, prevent additional pressure on terrestrial ecosystems, and revitalize rural areas. Likewise, growing perennial bioenergy crops, like miscanthus, for bioenergy production on abandoned cropland has emerged as a more sustainable approach to strategically expand bioenergy supply and revitalize rural areas at reduced risks for land competition, food security and environmental stress^74–76^. However, it is also essential to recognize that there are also important limitations such as potential future competition for biomass use in other sectors that are also projecting an energy transition (e.g., steel, bricks, and chemical industries), as well as biomass procurement and logistics challenges and costs^71^. Major transitions projected for the land use and energy sectors at a global level can significantly increase sustainable biomass resource availability^77,78^. Therefore, a sustainable supply of biomass from international markets might be key to realize the expected climate change mitigation levels in the many industrial sectors.

## Conclusions

The retrofitting of cement plants with oxyfuel capture technologies can provide significant reductions in the climate change impacts. The use of this CCS technology in combination with increasing use of alternative fuels with high biogenic shares, such as biomass from forest residues or dedicated bioenergy crops like miscanthus, allows achieving negative emissions in the cement clinker production process. However, the increased use of electricity in both air separation and carbon purification units in the oxyfuel technology causes trade-offs in other environmental impact categories, such as increased impacts in depletion of fossil and water resources and human toxicity. Similarly, the availability of biomass resources is likely to be limited and their sustainable supply needs to be secured. There is potential in existing biomass residues streams or suboptimal agricultural practices, but the competition for these feedstocks is likely to increase in the future. If sustainable biomass supply is not available at the scale that will be needed, the cement production sector cannot achieve large-scale negative CO_2_ emissions. Improved biomass certification schemes are instrumental to secure the sustainable supply of the different biomass resource options given the capped markets from residues and growing demand for biomass resources from the many industrial sectors facing an energy transition. Consequently, measures should be in place to prevent that the achievement of carbon negative emission technologies cause a rebound effect of larger cement production and use, as it may exacerbate resource consumption and competition and increase environmental impacts in other categories.

The implementation of future background inventories taking into consideration the projected trajectories for the energy sector and implementation of climate mitigation polices have a key role for the oxyfuel capture technology in the cement plants. Impacts from the cement clinker production process may decrease by more than 50% in 2050 when background inventories for the electricity systems consider the projected changes for the electricity sector. However, the magnitude of these changes depends on the context of the specific countries, considering current and future electricity mixes and intended climate targets to be achieved.

Large-scale implementation of mitigation options in the cement sector highly benefits from early-stage assessments considering the specific context, real-world operational data and boundary conditions from the different cement plants. Future refining and developments in the environmental implications of the large-scale adoption of the oxyfuel capture technology in combination with site-specific availability of biomass resources will be instrumental to identify, manage and prevent potential conflicting implications of the various relevant environmental impact categories.

## Supplementary Information

Supplementary Information 1.

Supplementary Information 2.

## Data Availability

Source data are provided with this paper. Source data for Figures are provided as Extended Data. Some of the datasets used in this analysis are publicly available from the references provided within the paper. Other data supporting the findings of this study are available from the corresponding author on reasonable request.

## References

[1] Churkina G (2020). Buildings as a global carbon sink. Nat. Sustain..

[2] Monteiro PJM, Miller SA, Horvath A (2017). Towards sustainable concrete. Nat. Mater..

[3] Miller SA, Moore FC (2020). Climate and health damages from global concrete production. Nat. Clim. Chang..

[4] International Energy Agency. *Technology Roadmap: Low-Carbon Transition in the Cement Industry*. (2018).

[5] Vatopoulos K, Tzimas E (2012). Assessment of CO2 capture technologies in cement manufacturing process. J. Clean. Prod..

[6] Moya JA, Pardo N, Mercier A (2011). The potential for improvements in energy efficiency and CO2 emissions in the EU27 cement industry and the relationship with the capital budgeting decision criteria. J. Clean. Prod..

[7] Rahman A, Rasul MG, Khan MMK, Sharma S (2015). Recent development on the uses of alternative fuels in cement manufacturing process. Fuel.

[8] Miller SA, Horvath A, Monteiro PJM (2016). Readily implementable techniques can cut annual CO2 emissions from the production of concrete by over 20%. Environ. Res. Lett..

[9] Habert G (2020). Environmental impacts and decarbonization strategies in the cement and concrete industries. Nat. Rev. Earth Environ..

[10] Hills T, Leeson D, Florin N, Fennell P (2016). Carbon capture in the cement industry: Technologies, progress, and retrofitting. Environ. Sci. Technol..

[11] CEMBUREAU. *Cementing the European Green Deal, The European Cement Association*. (2020).

[12] Plaza MG, Martínez S, Rubiera F (2020). CO2 capture, use, and storage in the cement industry: State of the art and expectations. Energies.

[13] Preston, F. & Lehne, J. *Making concrete change. Innovation in low-carbon cement and concrete. Chatham House Report* (2018).

[14] Voldsund M (2019). Comparison of technologies for CO2 capture from cement production—Part 1: Technical evaluation. Energies.

[15] Ditaranto M, Bakken J (2019). Study of a full scale oxy-fuel cement rotary kiln. Int. J. Greenh. Gas Control.

[16] De Lena E (2017). Process integration study of tail-end Ca-Looping process for CO2 capture in cement plants. Int. J. Greenh. Gas Control.

[17] Míguez JL, Porteiro J, Pérez-Orozco R, Gómez MÁ (2018). Technology evolution in membrane-based CCS. Energies.

[18] Hills, T. P., Sceats, M. G. & Fennell, P. S. Chapter 10 Applications of CCS in the cement industry. in *Carbon Capture Storage. *The Royal Society of Chemistry, 2020: pp. 315–352. https://doi.org/10.1039/9781788012744-00315.

[19] Carrasco-Maldonado F (2016). Oxy-fuel combustion technology for cement production–state of the art research and technology development. Int. J. Greenh. Gas Control.

[20] Kroumian, C. *et al.* Description of the work and preliminary results of the AC2OCEM project in facilitating carbon capture technology in the cement industry using oxyfuel combustion. in *TCCS-11 - Trondheim Conference on CO2 Capture, Transport and Storage* (2021).

[21] Miller SA, Horvath A, Monteiro PJM (2018). Impacts of booming concrete production on water resources worldwide. Nat. Sustain..

[22] Carrasco F, Grathwohl S, Maier J, Ruppert J, Scheffknecht G (2019). Experimental investigations of oxyfuel burner for cement production application. Fuel.

[23] Gardarsdottir SO (2019). Comparison of technologies for CO2 capture from cement production—Part 2: Cost analysis. Energies.

[24] Li J, Tharakan P, Macdonald D, Liang X (2013). Technological, economic and financial prospects of carbon dioxide capture in the cement industry. Energy Policy.

[25] Voldsund, M. *et al.* CEMCAP Comparative techno-economic analysis of CO2 capture in cement plants (D4. 6). 2018. (2018).

[26] Rodríguez N, Murillo R, Abanades JC (2012). CO2 capture from cement plants using oxyfired precalcination and/or calcium looping. Environ. Sci. Technol..

[27] Yang F, Meerman JC, Faaij APC (2021). Carbon capture and biomass in industry: A techno-economic analysis and comparison of negative emission options. Renew. Sustain. Energy Rev..

[28] Hoenig, V., Hoppe, H., Koring, K. & Lemke, J. *ECRA CCS Project–Report on Phase III*. (2012).

[29] Rolfe A (2018). Technical and environmental study of calcium carbonate looping versus oxy-fuel options for low CO2 emission cement plants. Int. J. Greenh. Gas Control.

[30] Gerbelová H, Van Der Spek M, Schakel W (2017). Feasibility assessment of CO2 capture retrofitted to an existing cement plant: Post-combustion vs. oxy-fuel combustion technology. Energy Proc..

[31] Hellweg S, Milà I Canals L (2014). Emerging approaches, challenges and opportunities in life cycle assessment. Science.

[32] Sonnemann, G. *et al.* Life cycle thinking and the use of LCA in policies around the world. in Hauschild, M., Rosenbaum, R., & Olsen, S. (Eds.), *Life Cycle Assessment,* Springer, Cham, Switzerland (2018), pp. 429-463.

[33] Sala S, Amadei AM, Beylot A, Ardente F (2021). The evolution of life cycle assessment in European policies over three decades. Int. J. Life Cycle Assess..

[34] Wernet G (2016). The ecoinvent database version 3 (part I): overview and methodology. Int. J. Life Cycle Assess..

[35] Levasseur A (2016). Enhancing life cycle impact assessment from climate science: Review of recent findings and recommendations for application to LCA. Ecol. Indic..

[36] Cherubini F (2016). Bridging the gap between impact assessment methods and climate science. Environ. Sci. Policy.

[37] Levasseur, A. *et al.* Greenhouse gas emissions and climate change impacts. in Frischknecht R. & Jolliet O. (Eds.) *Global guidance for life cycle impact assessment indicators,* vol 1. United Nations Environment Programme, Nairobi.

[38] Tanaka, K., Cavalett, O., Collins, W. J. & Cherubini, F. Asserting the climate benefits of the coal-to-gas shift across temporal and spatial scales. *Nat. Clim. Chang.* **9**, 389-396 (2019).

[39] Joos F (2013). Carbon dioxide and climate impulse response functions for the computation of greenhouse gas metrics: A multi-model analysis. Atmos. Chem. Phys..

[40] Collins WJ (2013). Global and regional temperature-change potentials for near-term climate forcers. Atmos. Chem. Phys..

[41] Allen MR (2016). New use of global warming potentials to compare cumulative and short-lived climate pollutants. Nat. Clim. Chang..

[42] Myhre, G. *et al.* Anthropogenic and natural radiative forcing. *climate change 2013: The physical science basis. Contribution of working group I to the Fifth assessment report of the intergovernmental panel on climate change (eds. Stocker, T.F. et al.) Ch. 8, 659–740 (Cambridge University Press, 2013)* (2013).

[43] Huijbregts MAJ (2017). ReCiPe2016: a harmonised life cycle impact assessment method at midpoint and endpoint level. Int. J. Life Cycle Assess..

[44] Locher G (2002). Mathematical models for the cement clinker burning process, part 1: Reactions and unit operations. ZKG Int..

[45] Locher G (2002). Mathematical models for the cement clinker burning process Part 2: Preheater, calciner and bypass. ZKG Int..

[46] Locher G (2002). Mathematical models for the cement clinker burning process Part 3: Rotary kiln. ZKG Int..

[47] Locher G (2002). Mathematical models for the cement clinker burning process Part 4: Grate cooler. ZKG Int..

[48] Locher G (2002). Mathematical models for the cement clinker burning process-Part 5: Complete plant. ZKG Int..

[49] Koring, K. CO2 - Emissionsminderungspotential und technologische Auswirkungen der Oxyfuel-Technologie im Zementklinkerbrennprozess. (Verl. Bau + Technik, 2012).

[50] AC2OCEM. AC2OCEM Project. http://www.act-ccs.eu/ac2ocem (2021).

[51] Jamali, A., Fleiger, K., Ruppert, J., Hoenig, V. & Anantharaman, R. Optimised Opearation of an Oxyfuel Cement Plant (D6.1). (2018).

[52] ECRA, E. CCS Project-Report about phase II. *ECRA (European Cem. Res. Acad. Duesseldorf, Ger.* (2009).

[53] CSI/ECRA. *Development of State of the Art-Techniques in Cement Manufacturing: Trying to Look Ahead*. http://www.wbcsdcement.org/technology (2017).

[54] Jakobsen J, Roussanaly S, Anantharaman R (2017). A techno-economic case study of CO2 capture, transport and storage chain from a cement plant in Norway. J. Clean. Prod..

[55] Furre A-K, Meneguolo R, Ringrose P, Kassold S (2019). Building confidence in CCS: from sleipner to the northern lights project. First Break.

[56] CSI/GCCA. *Getting the Numbers Right. Emissions Report 2019*. (2019).

[57] Schakel W (2018). Impact of fuel selection on the environmental performance of post-combustion calcium looping applied to a cement plant. Appl. Energy.

[58] Murphy F, Devlin G, McDonnell K (2013). Miscanthus production and processing in Ireland: An analysis of energy requirements and environmental impacts. Renew. Sustain. Energy Rev..

[59] de Jong J, Akselsson C, Egnell G, Löfgren S, Olsson BA (2017). Realizing the energy potential of forest biomass in Sweden-How much is environmentally sustainable?. For. Ecol. Manage..

[60] Lundmark T (2014). Potential roles of Swedish forestry in the context of climate change mitigation. Forests.

[61] Cavalett O, Cherubini F (2018). Contribution of jet fuel from forest residues to multiple Sustainable Development Goals. Nat. Sustain..

[62] Joyce, P. J. & Björklund, A. Futura: A new tool for transparent and shareable scenario analysis in prospective life cycle assessment. *J. Ind. Ecol. ***26**, 134-144 (2022).

[63] Luderer G (2019). Environmental co-benefits and adverse side-effects of alternative power sector decarbonization strategies. Nat. Commun..

[64] Mendoza Beltran A (2020). When the background matters: Using scenarios from integrated assessment models in prospective life cycle assessment. J. Ind. Ecol..

[65] Sacchi R (2022). PRospective EnvironMental Impact asSEment (premise): A streamlined approach to producing databases for prospective life cycle assessment using integrated assessment models. Renew. Sustain. Energy Rev..

[66] Baumstark, L. *et al.* REMIND2. 1: Transformation and innovation dynamics of the energy-economic system within climate and sustainability limits. *Geosci. Model Dev. ***14**(10), 6571–6603 (2021).

[67] Fricko O (2017). The marker quantification of the Shared Socioeconomic Pathway 2: A middle-of-the-road scenario for the 21st century. Glob. Environ. Chang..

[68] Pregger T, Nitsch J, Naegler T (2013). Long-term scenarios and strategies for the deployment of renewable energies in Germany. Energy Policy.

[69] Luca de Tena D, Pregger T (2018). Impact of electric vehicles on a future renewable energy-based power system in Europe with a focus on Germany. Int. J. Energy Res..

[70] Millot A, Krook-Riekkola A, Maïzi N (2020). Guiding the future energy transition to net-zero emissions: Lessons from exploring the differences between France and Sweden. Energy Policy.

[71] Mikulčić H, Klemeš JJ, Vujanović M, Urbaniec K, Duić N (2016). Reducing greenhouse gasses emissions by fostering the deployment of alternative raw materials and energy sources in the cleaner cement manufacturing process. J. Clean. Prod..

[72] Thorenz A, Wietschel L, Stindt D, Tuma A (2018). Assessment of agroforestry residue potentials for the bioeconomy in the European Union. J. Clean. Prod..

[73] Camia, A. *et al.* The use of woody biomass for energy production in the EU, EUR 30548 EN, Publications Office of the European Union, Luxembourg (2020).

[74] Campbell JE, Lobell DB, Genova RC, Field CB (2008). The global potential of bioenergy on abandoned agriculture lands. Environ. Sci. Technol..

[75] Næss JS, Cavalett O, Cherubini F (2021). The land–energy–water nexus of global bioenergy potentials from abandoned cropland. Nat. Sustain..

[76] Robertson GP (2017). Cellulosic biofuel contributions to a sustainable energy future: Choices and outcomes. Science.

[77] Popp A (2017). Land-use futures in the shared socio-economic pathways. Glob. Environ. Chang..

[78] IPCC, 2019: Climate Change and Land: an IPCC special report on climate change, desertification, land degradation, sustainable land management, food security, and greenhouse gas fluxes in terrestrial ecosystems [Shukla, P. R. *et al. *(eds.)]. In press.

