# Supplementary material for: LCA and negative emission potential of retrofitted cement plants under oxyfuel conditions at high biogenic fuel shares
Source: Sci Rep. 2022 May 27;12:8924. doi: 10.1038/s41598-022-13064-w (PMC9142509; doi:10.1038/s41598-022-13064-w)
Supplement: Supplementary file 2 — Supplementary Information 2. [file 41598_2022_13064_MOESM2_ESM.docx]

**Supplementary Information**

**for:**

**“LCA and negative emission potential of retrofitted cement plants under oxyfuel conditions at high biogenic fuel shares”**

**Otavio Cavalett^a^*, Marcos D. B. Watanabe^a^, Kristina Fleiger**^b^**, Volker Hoenig^b^ Francesco Cherubini^a^**

^a^Department of Energy and Process Engineering, Industrial Ecology Programme, Norwegian University of Science and Technology (NTNU), Høgskoleringen 1, 7491, Trondheim, Norway

^b^VDZ gGmbH, Toulouser Allee 71, 40476 Düsseldorf, Germany

*Corresponding Author, e-mail: otavio.cavalett@ntnu.no

**
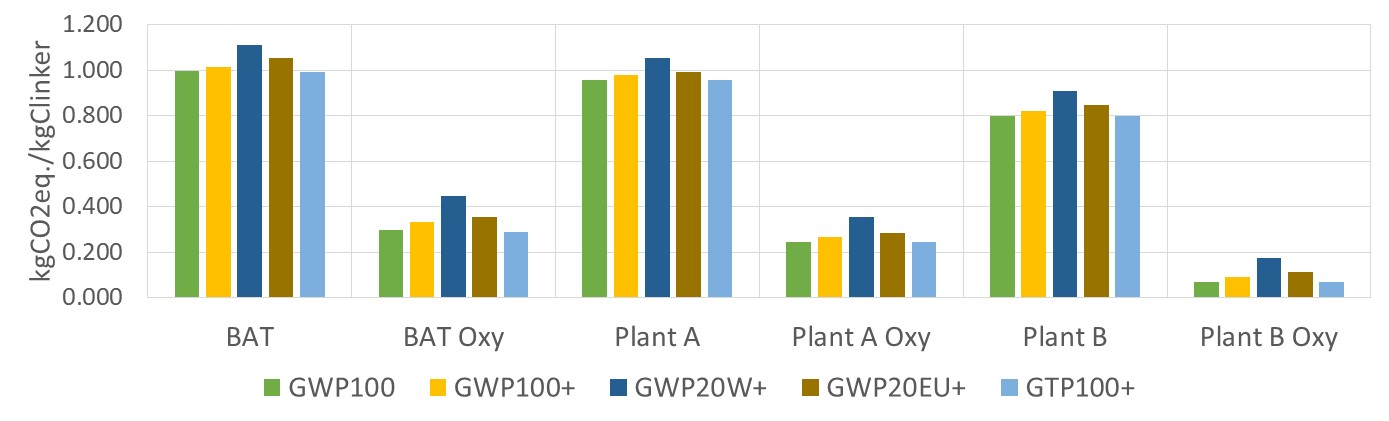
**

**Supplementary Fig. 1.** Climate change impacts categories cement clinker production cases under conventional and oxyfuel CCS benchmarked against REF process using different climate metric and time horizons. Climate metrics with “+” signal include contributions of near-term climate forcers (NTCF) in addition to well mixed GHGs. GTP100 is Global Temperature Potential at the time horizon of 100 years and GWP 20 and GWP 100 in the Global Warming Potential for 20 and 100 years respectively. GWP 20EU and GWP20W indicate that metric for near term climate forcers are used considering climate metrics for emission occurring in Europe and world averaged metrics, respectively.

**Supplementary Table 1:** Characterization factors for the climate change impacts.

| Emission | GWP100 | GWP100+ | GWP20W+ | GWP20EU+ | GTP100+ |
| --- | --- | --- | --- | --- | --- |
| Carbon dioxide, fossil | 1 | 1 | 1 | 1 | 1 |
| Carbon dioxide, biogenic | 1 | 1 | 1 | 1 | 1 |
| Carbon monoxide, fossil |  | 2.1 | 7.8 | 4.9 | -0.2 |
| Carbon monoxide, biogenic |  | 2.1 | 7.8 | 4.9 | -0.2 |
| Methane, biogenic | 22.3 | 22.3 | 86 | 86 | 11 |
| Methane, fossil | 25 | 25 | 87 | 87 | 13 |
| Dinitrogen monoxide | 298 | 298 | 268 | 268 | 297 |
| Nitrogen oxides |  | -10.7 | -40.0 | -14.6 | -0.7 |
| Sulfur dioxide |  | -38 | -141 | -141 | -5 |
| NMVOC, non-methane volatile organic compound |  | 5.5 | 18.7 | 18 | 0.8 |
| VOC, volatile organic compounds |  | 5.5 | 18.7 | 18 | 0.8 |
| Organic carbon |  | -43 | -160 | -172 | -6.7 |
| Black carbon |  | 846 | 3200 | 1480 | 120 |

**Note:** Climate metrics with “+” signal include contributions of near-term climate forcers (NTCF) in addition to well mixed GHGs. GTP100 is Global Temperature Potential at the time horizon of 100 years and GWP 20 and GWP 100 in the Global Warming Potential for 20 and 100 years respectively. GWP 20EU and GWP20W indicate that metric for near term climate forcers are used considering climate metrics for emission occurring in Europe and world averaged metrics, respectively.

**Supplementary Table 2:** Proprieties of conventional and alternative fuels used in the cement plants. Data from industry databank, except when indicated.

| Alternative fuels | Carbon content  (kgC/kgfuel_db_) | Biogenic carbon share^a^  (%) | Lower heating value  (MJ/kg) | Moisture content  (%) |
| --- | --- | --- | --- | --- |
| Lignite | 0.637 | 0 | 21.39 | 10 |
| Hard coal | 0.696 | 0 | 26.42 | 9 |
| Waste oil | 0.733 | 0 | 35.23 | 6 |
| Roofing felt | 0.484 | 0 | 18.73 | 9 |
| Destillation residues | 0.442 | 0 | 14.67 | 22 |
| Used tyres | 0.686 | 27 | 29.00 | 6 |
| RDF, type ‘a’ | 0.588 | 30 | 22.99 | 13 |
| RDF, type ‘b’ | 0.389 | 0 | 12.81 | 21 |
| RDF, type ‘c’ | 0.565 | 30 | 20.79 | 13 |
| RDF, type ‘d’ | 0.461 | 30 | 16.30 | 17 |
| RDF, type ‘e’ | 0.533 | 30 | 22.01 | 26 |
| Biomass, miscanthus^b^ | 0.455 | 100 | 16.63 | 9.5 |
| Biomass, forest residues^c^ | 0.500 | 100 | 16.50 | 33.3 |

^a^ ref.^1^; ^b^ ref.^2^; ^c^ ref.^3^;

**Supplementary Table 3:** Life cycle inventory for plant A cases.

|  | Plant A | Plant A Oxy | Plant A B(M) | Plant A OxyB(M) | Plant A OxyB(M)+ | Plant A B(FR) | Plant A OxyB(FR) | Plant A OxyB(FR)+ |
| --- | --- | --- | --- | --- | --- | --- | --- | --- |
| Inputs form technosphere |  |  |  |  |  |  |  |  |
| Lime | 1.078 | 1.078 | 1.078 | 1.078 | 1.078 | 1.078 | 1.078 | 1.078 |
| Clay | 0.062 | 0.062 | 0.062 | 0.062 | 0.062 | 0.062 | 0.062 | 0.062 |
| Sand | 0.159 | 0.159 | 0.159 | 0.159 | 0.159 | 0.159 | 0.159 | 0.159 |
| Hard coal |  |  |  |  |  |  |  |  |
| Lignite | 0.0173 | 0.0183 | 0.0128 | 0.0135 |  | 0.0128 | 0.0135 |  |
| RDF, type ‘a’ | 0.0432 | 0.0458 | 0.0320 | 0.0339 | 0.0339 | 0.0320 | 0.0339 | 0.0339 |
| waste oil | 0.0049 | 0.0051 | 0.0036 | 0.0038 | 0.0038 | 0.0036 | 0.0038 | 0.0038 |
| Roofing felt | 0.0243 | 0.0000 |  |  |  |  |  |  |
| Destillation residues | 0.0324 | 0.0257 |  |  |  |  |  |  |
| RDF, type ‘b’ | 0.0216 | 0.0343 |  |  |  |  |  |  |
| RDF, type ‘c’ | 0.0216 | 0.0229 |  |  |  |  |  |  |
| RDF, type ‘d’ | 0.0541 | 0.0229 |  |  |  |  |  |  |
| Used tyres |  |  |  |  |  |  |  |  |
| RDF, type ‘e’ |  |  |  |  |  |  |  |  |
| Biomass, miscanthus (db) |  |  | 0.1917 | 0.2020 | 0.2195 |  |  |  |
| Biomass, wood chips (db) |  |  |  |  |  | 0.1932 | 0.2036 | 0.2212 |
| Transport, freight, lorry 16-32ton | 0.26 | 0.245 | 0.076 | 0.081 | 0.067 | 0.084 | 0.089 | 0.076 |
| Transport, freight, sea, bulk carrier for dry goods |  |  |  |  |  |  |  |  |
| Electricity, European mix |  |  |  |  |  |  |  |  |
| Electricity, German mix | 0.042 | 0.251 | 0.045 | 0.248 | 0.247 | 0.045 | 0.248 | 0.247 |
| Electricity, Swedish mix |  |  |  |  |  |  |  |  |
| Water | 0.34 | 0.34 | 0.34 | 0.34 | 0.34 | 0.34 | 0.34 | 0.34 |
| Ammonia, liquid | 0.0050 | 0.0050 | 0.0050 | 0.0050 | 0.0050 | 0.0050 | 0.0050 | 0.0050 |
| Emissions to air |  |  |  |  |  |  |  |  |
| CO_2_ from calcinaiton | 0.451 | 0.045 | 0.451 | 0.045 | 0.045 | 0.451 | 0.045 | 0.045 |
| CO_2_ biogenic from non-biomass fuels | 0.080 | -0.076 | 0.024 | -0.023 | -0.017 | 0.024 | -0.023 | -0.017 |
| CO_2_ fossil from fuels | 0.396 | 0.042 | 0.101 | 0.011 | 0.008 | 0.101 | 0.011 | 0.008 |
| CO_2_ biogenic from biomass fuels |  |  | 0.314 | -0.297 | -0.323 | 0.347 | -0.329 | -0.358 |
| Carbom monoxide | 5.13E-04 | 5.13E-05 | 5.13E-04 | 5.13E-05 | 5.13E-05 | 5.13E-04 | 5.13E-05 | 5.13E-05 |
| Nitogen oxides | 3.42E-04 | 3.42E-05 | 3.42E-04 | 3.42E-05 | 3.42E-05 | 3.42E-04 | 3.42E-05 | 3.42E-05 |
| Sulfur oxides | 1.45E-05 | 1.45E-06 | 1.45E-05 | 1.45E-06 | 1.45E-06 | 1.45E-05 | 1.45E-06 | 1.45E-06 |
| NMVOC, non-methane volatile organic compound | 5.64E-05 | 5.64E-06 | 5.64E-05 | 5.64E-06 | 5.64E-06 | 5.64E-05 | 5.64E-06 | 5.64E-06 |
| Particulates, < 2.5 um | 1.82E-05 | 1.82E-05 | 1.82E-05 | 1.82E-05 | 1.82E-05 | 1.82E-05 | 1.82E-05 | 1.82E-05 |
| Particulates, > 10 um | 4.27E-06 | 4.27E-06 | 4.27E-06 | 4.27E-06 | 4.27E-06 | 4.27E-06 | 4.27E-06 | 4.27E-06 |
| Particulates, > 2.5 um, and < 10um | 5.98E-06 | 5.98E-06 | 5.98E-06 | 5.98E-06 | 5.98E-06 | 5.98E-06 | 5.98E-06 | 5.98E-06 |
| Particulates, unspecified |  |  |  |  |  |  |  |  |
| Ammonia | 2.28E-05 | 2.28E-06 | 2.28E-05 | 2.28E-06 | 2.28E-06 | 2.28E-05 | 2.28E-06 | 2.28E-06 |
| Water, air | 3.40E-04 | 3.40E-04 | 3.40E-04 | 3.40E-04 | 3.40E-04 | 3.40E-04 | 3.40E-04 | 3.40E-04 |

**Supplementary Table 4:** Life cycle inventory for plant B cases.

|  | Plant B | Plant B Oxy | Plant B B(M) | Plant B OxyB(M) | Plant B OxyB(M)+ | Plant B B(FR) | Plant B OxyB(FR) | Plant B OxyB(FR)+ |
| --- | --- | --- | --- | --- | --- | --- | --- | --- |
| Inputs form technosphere |  |  |  |  |  |  |  |  |
| Lime | 0.900 | 0.900 | 0.900 | 0.900 | 0.900 | 0.900 | 0.900 | 0.900 |
| Clay | 0.115 | 0.115 | 0.115 | 0.115 | 0.115 | 0.115 | 0.115 | 0.115 |
| Sand | 0.151 | 0.151 | 0.151 | 0.151 | 0.151 | 0.151 | 0.151 | 0.151 |
| Hard coal | 0.0292 | 0.0291 | 0.0149 | 0.0149 | 0.0000 | 0.0152 | 0.0151 | 0.0000 |
| Lignite |  |  |  |  |  |  |  |  |
| RDF, type ‘a’ | 0.0530 | 0.0528 | 0.0372 | 0.0332 | 0.0332 | 0.0378 | 0.0338 | 0.0338 |
| waste oil |  |  |  |  |  |  |  |  |
| Roofing felt |  |  |  |  |  |  |  |  |
| Destillation residues |  |  |  |  |  |  |  |  |
| RDF, type ‘b’ |  |  |  |  |  |  |  |  |
| RDF, type ‘c’ |  |  |  |  |  |  |  |  |
| RDF, type ‘d’ |  |  |  |  |  |  |  |  |
| Used tyres | 0.0090 | 0.0089 |  |  |  |  |  |  |
| RDF, type ‘e’ | 0.0430 | 0.0428 |  |  |  |  |  |  |
| Biomass, miscanthus (db) |  |  | 0.1520 | 0.1357 | 0.1569 |  |  |  |
| Biomass, wood chips (db) |  |  |  |  |  | 0.1519 | 0.1356 | 0.1573 |
| Transport, freight, lorry 16-32ton | 0.17 | 0.17 | 0.07 | 0.07 | 0.06 | 0.08 | 0.08 | 0.07 |
| Transport, freight, sea, bulk carrier for dry goods | 0.09 | 0.09 | 0.03 | 0.03 | 0.02 | 0.03 | 0.03 | 0.02 |
| Electricity, European mix |  |  |  |  |  |  |  |  |
| Electricity, German mix |  |  |  |  |  |  |  |  |
| Electricity, Swedish mix | 0.104 | 0.278 | 0.107 | 0.271 | 0.269 | 0.107 | 0.275 | 0.273 |
| Water | 0.34 | 0.34 | 0.34 | 0.34 | 0.34 | 0.34 | 0.34 | 0.34 |
| Ammonia, liquid | 0.0050 | 0.0050 | 0.0050 | 0.0050 | 0.0050 | 0.0050 | 0.0050 | 0.0050 |
| Emissions to air |  |  |  |  |  |  |  |  |
| CO_2_ from calcinaiton | 0.376 | 0.038 | 0.376 | 0.038 | 0.038 | 0.376 | 0.038 | 0.038 |
| CO_2_ biogenic from non-biomass fuels | 0.100 | -0.089 | 0.038 | -0.027 | -0.028 | 0.039 | -0.027 | -0.029 |
| CO_2_ fossil from fuels | 0.343 | 0.034 | 0.113 | 0.012 | 0.007 | 0.115 | 0.012 | 0.007 |
| CO_2_ biogenic from biomass fuels |  |  | 0.249 | -0.200 | -0.231 | 0.273 | -0.200 | -0.254 |
| Carbom monoxide | 2.39E-03 | 2.39E-04 | 2.39E-03 | 2.39E-04 | 2.39E-04 | 2.39E-03 | 2.39E-04 | 2.39E-04 |
| Nitogen oxides | 1.59E-04 | 1.59E-05 | 1.59E-04 | 1.59E-05 | 1.59E-05 | 1.59E-04 | 1.59E-05 | 1.59E-05 |
| Sulfur oxides | 1.59E-05 | 1.59E-06 | 1.59E-05 | 1.59E-06 | 1.59E-06 | 1.59E-05 | 1.59E-06 | 1.59E-06 |
| NMVOC, non-methane volatile organic compound | 5.64E-05 | 5.64E-06 | 5.64E-05 | 5.64E-06 | 5.64E-06 | 5.64E-05 | 5.64E-06 | 5.64E-06 |
| Particulates, < 2.5 um | 1.64E-05 | 1.64E-05 | 1.64E-05 | 1.64E-05 | 1.64E-05 | 1.64E-05 | 1.64E-05 | 1.64E-05 |
| Particulates, > 10 um | 3.84E-06 | 3.84E-06 | 3.84E-06 | 3.84E-06 | 3.84E-06 | 3.84E-06 | 3.84E-06 | 3.84E-06 |
| Particulates, > 2.5 um, and < 10um | 5.38E-06 | 5.38E-06 | 5.38E-06 | 5.38E-06 | 5.38E-06 | 5.38E-06 | 5.38E-06 | 5.38E-06 |
| Particulates, unspecified |  |  |  |  |  |  |  |  |
| Ammonia | 4.94E-06 | 4.94E-07 | 4.94E-06 | 4.94E-07 | 4.94E-07 | 4.94E-06 | 4.94E-07 | 4.94E-07 |
| Water, air | 3.40E-04 | 3.40E-04 | 3.40E-04 | 3.40E-04 | 3.40E-04 | 3.40E-04 | 3.40E-04 | 3.40E-04 |

**Supplementary Table 5:** Life cycle inventory for the REF cases.

|  | REF | REF Oxy |
| --- | --- | --- |
| Inputs form technosphere |  |  |
| Lime | 1.350 | 1.303 |
| Clay | 0.128 | 0.123 |
| Sand | 0.229 | 0.221 |
| Hard coal | 0.1150 | 0.1160 |
| Lignite |  |  |
| RDF, type ‘a’ |  |  |
| waste oil |  |  |
| Roofing felt |  |  |
| Destillation residues |  |  |
| RDF, type ‘b’ |  |  |
| RDF, type ‘c’ |  |  |
| RDF, type ‘d’ |  |  |
| Used tyres |  |  |
| RDF, type ‘e’ |  |  |
| Biomass, miscanthus (db) |  |  |
| Biomass, wood chips (db) |  |  |
| Transport, freight, lorry 16-32ton | 0.064 | 0.064 |
| Transport, freight, sea, bulk carrier for dry goods |  |  |
| Electricity, European mix | 0.132 | 0.281 |
| Electricity, German mix |  |  |
| Electricity, Swedish mix |  |  |
| Water | 0.34 | 0.34 |
| Ammonia, liquid | 0.0050 | 0.0050 |
| Emissions to air |  |  |
| CO_2_ from calcinaiton | 0.543 | 0.056 |
| CO_2_ biogenic from non-biomass fuels |  |  |
| CO_2_ fossil from fuels | 0.307 | 0.032 |
| CO_2_ biogenic from biomass fuels |  |  |
| Carbom monoxide | 1.70E-03 | 1.70E-04 |
| Nitogen oxides | 1.01E-03 | 1.01E-04 |
| Sulfur oxides | 4.02E-04 | 4.02E-05 |
| NMVOC, non-methane volatile organic compound | 5.64E-05 | 5.64E-06 |
| Particulates, < 2.5 um |  |  |
| Particulates, > 10 um |  |  |
| Particulates, > 2.5 um, and < 10um |  |  |
| Particulates, unspecified | 1.70E-05 | 1.70E-06 |
| Ammonia | 2.28E-05 | 2.28E-06 |
| Water, air | 3.40E-04 | 3.40E-04 |

**Supplementary Table 6:** Details about of the cement production cases considered in this study.

| Cement production case | Description | Retrofitted to oxyfuel technology | Fuel mix | Lower heating value of the fuel mix  (MJ klg^-1^) | Life cycle inventory data based on |
| --- | --- | --- | --- | --- | --- |
| REF | Cement production considering modern process technology based on the average cement sector in Europe | no | 100% hard coal | 26.42 | (CSI/ECRA, 2017; Gardarsdottir et al., 2019; Voldsund et al., 2019) |
| REF Oxy | Same as REF but considering the plant retrofitted to oxyfuel operational conditions | yes | 100% hard coal | 26.42 | 2017; Voldsund et al. 2019; Gardarsdottir et al. 2019) |
| Plant A | Plant A operating under current (real world) operational conditions | no | 9% lignite  91% alternative fuels (based on RDF with a mix with 17% biogenic sources) | 18.57 | Plant operational data |
| Plant A Oxy | Plant A retrofitted to oxyfuel operational conditions | yes | 9% lignite  91% alternative fuels (based on RDF with a mix with 17% biogenic sources) | 18.80 | Plant operational data in combination to process simulation |
| Plant A B(M) | Plant A operating under conventional (air) conditions and a higher share of biomass from miscanthus as alternative fuels | no | 6% lignite  74% biomass from miscanthus  20% other alternative fuels (based on RDF with a mix with 19% biogenic sources) | 18.01 | Plant operational data in combination to process simulation |
| Plant A OxyB(M) | Plant A retrofitted to oxyfuel operational conditions using a higher share of biomass from miscanthus as alternative fuels | yes | 6% lignite  74% biomass from miscanthus  20% other alternative fuels (based on RDF with a mix with 19% biogenic sources) | 18.01 | Plant operational data in combination to process simulation |
| Plant A OxyB(M)+ | Plant A retrofitted to oxyfuel operational conditions using 100% biomass from miscanthus as alternative fuel. | yes | 80% biomass from miscanthus  20% other alternative fuels (based on RDF with a mix with 19% biogenic sources) | 17.74 | Plant operational data in combination to process simulation |
| Plant A B(FR) | Plant A operating under conventional (air) conditions and a higher share of biomass from forest residues as alternative fuels | no | 6% lignite  74% biomass from miscanthus  20% other alternative fuels (based on RDF with a mix with 19% biogenic sources) | 17.90 | Plant operational data in combination to process simulation |
| Plant A OxyB(FR) | Plant A retrofitted to oxyfuel operational conditions using a higher share of biomass from forest residues as alternative fuels | yes | 6% lignite  74% biomass from forest residues  20% other alternative fuels (based on RDF with a mix with 19% biogenic sources) | 17.90 | Plant operational data in combination to process simulation |
| Plant A OxyB(FR)+ | Plant A retrofitted to oxyfuel operational conditions using 100% biomass from forest residues as alternative fuel | yes | 80% biomass from forest residues  20% other alternative fuels (based on RDF with a mix with 19% biogenic sources) | 17.62 | Plant operational data in combination to process simulation |
| Plant B | Plant B operating under current (real world) operational conditions | no | 22% hard coal  78% alternative fuels (based on RDF with a mix with 23% biogenic sources) | 23.82 | Plant operational data |
| Plant B Oxy | Plant B retrofitted to oxyfuel operational conditions | yes | 22% hard coal  78% alternative fuels (based on RDF with a mix with 23% biogenic sources) | 23.82 | Plant operational data in combination to process simulation |
| Plant B B(M) | Plant B operating under conventional (air) conditions and a higher share of biomass from miscanthus as alternative fuels | no | 10% hard coal  66% biomass from miscanthus  24% other alternative fuels (based on RDF with a mix with 25% biogenic sources) | 18.51 | Plant operational data in combination to process simulation |
| Plant B OxyB(M) | Plant B retrofitted to oxyfuel operational conditions using a higher share of biomass from miscanthus as alternative fuels | yes | 10% hard coal  66% biomass from miscanthus  24% other alternative fuels (based on RDF with a mix with 25% biogenic sources) | 18.57 | Plant operational data in combination to process simulation |
| Plant B OxyB(M)+ | Plant B retrofitted to oxyfuel operational conditions using 100% biomass from miscanthus as alternative fuel. | yes | 76% biomass from miscanthus  24% other alternative fuels (based on RDF with a mix with 30% biogenic sources) | 17.74 | Plant operational data in combination to process simulation |
| Plant B B(FR) | Plant B operating under conventional (air) conditions and a higher share of biomass from forest residues as alternative fuels | no | 10% hard coal  66% biomass from forest residues  24% other alternative fuels (based on RDF with a mix with 25% biogenic sources) | 18.43 | Plant operational data in combination to process simulation |
| Plant B OxyB(FR) | Plant B retrofitted to oxyfuel operational conditions using a higher share of biomass from forest residues as alternative fuels | yes | 10% hard coal  66% biomass from forest residues  24% other alternative fuels (based on RDF with a mix with 25% biogenic sources) | 18.50 | Plant operational data in combination to process simulation |
| Plant B OxyB(FR)+ | Plant B retrofitted to oxyfuel operational conditions using 100% biomass from forest residues as alternative fuel | yes | 76% biomass from forest residues  24% other alternative fuels (based on RDF with a mix with 30% biogenic sources) | 17.65 | Plant operational data in combination to process simulation |

**Supplementary Table 7:** Electricity mix for Germany up to 2050 considering projections aiming at the long-term gradual implementation of increased shares of renewable energy. Based on refs.^4,5^.

| Technology | 2030 | 2040 | 2050 |
| --- | --- | --- | --- |
| Photovoltaic | 14% | 14% | 15% |
| Wind onshore | 21% | 20% | 20% |
| Wind offshore | 14% | 18% | 21% |
| Biomass and biogas CHP | 11% | 12% | 12% |
| Hydropower | 4% | 5% | 5% |
| Geothermal | 1% | 2% | 3% |
| Imports (renewable) | 4% | 8% | 10% |
| Lignite | 7% | 3% | 2% |
| Hard coal | 7% | 3% | 0% |
| Gas turbines | 17% | 14% | 10% |
| Hydrogen | 0% | 0% | 2% |

**Supplementary Table 8:** Electricity mix for Sweden up to 2050 considering projections aiming at the long-term gradual implementation of increased shares of renewable energy. Based on Ref.^6^.

| Technology | 2030 | 2040 | 2050 |
| --- | --- | --- | --- |
| Nuclear | 17% | 6% | 0% |
| Hydropower | 32% | 25% | 19% |
| Oil | 1% | 0% | 0% |
| Gas | 4% | 2% | 0% |
| Biomass and biogas CHP | 25% | 11% | 5% |
| Wind onshore | 17% | 48% | 59% |
| Photovoltaic | 0% | 8% | 16% |
| Waste | 4% | 2% | 0% |

**Supplementary Table 9:** Fraction of climate impacts from non-CO_2_ climate forcers.

|  | GWP100 | GWP100+ | GWP20W+ | GWP20EU+ | GTP100+ |
| --- | --- | --- | --- | --- | --- |
| REF | 2% | 5% | 12% | 7% | 2% |
| REF Oxy | 9% | 13% | 18% | 39% | 24% |
| Plant A | 1% | 3% | 10% | 4% | 1% |
| Plant A Oxy | 5% | 15% | 35% | 18% | 5% |
| plant B | 1% | 4% | 13% | 7% | 1% |
| plant B Oxy | 14% | 38% | 66% | 47% | 14% |

**Note:** Climate metrics with “+” signal include contributions of near-term climate forcers (NTCF) in addition to well mixed GHGs. GTP100 is Global Temperature Potential at the time horizon of 100 years and GWP 20 and GWP 100 in the Global Warming Potential for 20 and 100 years respectively. GWP 20EU and GWP20W indicate that metric for near term climate forcers are used considering climate metrics for emission occurring in Europe and world averaged metrics, respectively.

**References**

1. Koring, K. *et al.* Deployment of CCS in the Cement Industry. *IEA Rep. 2013* **19**, (2013).

2. Samson, A., Mos, M., Najser, J., Daroch, M. & Gallagher, J. Gasification of Miscanthus x giganteus Pellets in a Fixed bed Pilot-scale Unit. *Front. Energy Res.* **6**, 91 (2018).

3. Benhelal, E., Zahedi, G., Shamsaei, E. & Bahadori, A. Global strategies and potentials to curb CO2 emissions in cement industry. *J. Clean. Prod.* **51**, 142–161 (2013).

4. Pregger, T., Nitsch, J. & Naegler, T. Long-term scenarios and strategies for the deployment of renewable energies in Germany. *Energy Policy* **59**, 350–360 (2013).

5. Luca de Tena, D. & Pregger, T. Impact of electric vehicles on a future renewable energy‐based power system in Europe with a focus on Germany. *Int. J. Energy Res.* **42**, 2670–2685 (2018).

6. Millot, A., Krook-Riekkola, A. & Maïzi, N. Guiding the future energy transition to net-zero emissions: Lessons from exploring the differences between France and Sweden. *Energy Policy* **139**, 111358 (2020).
